# Occurrence, Pathogenicity, and Mycotoxin Production of *Fusarium temperatum* in Relation to Other *Fusarium* Species on Maize in Germany

**DOI:** 10.3390/pathogens9110864

**Published:** 2020-10-22

**Authors:** Annette Pfordt, Simon Schiwek, Anna Rathgeb, Charlotte Rodemann, Nele Bollmann, Matthias Buchholz, Petr Karlovsky, Andreas von Tiedemann

**Affiliations:** 1 Plant Pathology and Crop Protection, University of Goettingen, 37077 Goettingen, Germany; annette.pfordt@uni-goettingen.de (A.P.); charlotte.rodemann@stud.uni-goettingen.de (C.R.); nele.bollmann@stud.uni-goettingen.de (N.B.); matthias.buchholz@uni-hohenheim.de (M.B.); 2 Molecular Phytopathology and Mycotoxin Research, University of Goettingen, 37077 Goettingen, Germany; simon.schiwek@uni-goettingen.de (S.S.); annarathgeb@gmail.com (A.R.)

**Keywords:** *Fusarium temperatum*, *Fusarium subglutinans*, pathogenicity, maize ear rot, *Fusarium* head blight, beauvericin, translation elongation factor *1α*

## Abstract

*Fusarium subglutinans* is a plant pathogenic fungus infecting cereal grain crops. In 2011, the species was divided in *Fusarium temperatum* *sp. nov.* and *F. subglutinans sensu stricto*. In order to determine the occurrence and significance of *F. temperatum* and *F. subglutinans* on maize, a monitoring of maize ears and stalks was carried out in Germany in 2017 and 2018. Species identification was conducted by analysis of the translation elongation factor *1α* (*TEF-1α*) gene. Ninety-four isolates of *F. temperatum* and eight isolates of *F. subglutinans* were obtained during two years of monitoring from 60 sampling sites in nine federal states of Germany. Inoculation of maize ears revealed a superior aggressiveness for *F. temperatum*, followed by *Fusarium graminearum*, *Fusarium verticillioides*, and *F. subglutinans.* On maize stalks, *F. graminearum* was the most aggressive species while *F. temperatum* and *F. subglutinans* caused only small lesions. The optimal temperature for infection of maize ears with *F. temperatum* was 24 °C and 21 °C for *F. subglutinans*. All strains of *F. temperatum* and *F. subglutinans* were pathogenic on wheat and capable to cause moderate to severe head blight symptoms. The assessment of mycotoxin production of 60 strains of *F. temperatum* cultivated on rice revealed that all strains produced beauvericin, moniliformin, fusaric acid, and fusaproliferin. The results demonstrate a higher prevalence and aggressiveness of *F. temperatum* compared to *F. subglutinans* in German maize cultivation areas.

## 1. Introduction

*Fusarium* ear and stalk rot are ubiquitous diseases of maize with high economic impact in agriculture [1]. Several *Fusarium* species infecting maize are known to produce toxic secondary metabolites, called mycotoxins, which impair grain quality and threaten the safety of animal feed and food products [2,3]. Among the most important *Fusarium* species inciting pre- and post-harvest ear rot of maize are *F. graminearum* and *F. verticillioides* [4,5,6], but also other species such as *Fusarium poae* [7,8], *Fusarium proliferatum* [1], *F. subglutinans* [9], and *F. temperatum* [10] are frequently reported. Ear infection is typically characterized by the growth of white or reddish mycelium with rot induced on the cob and on stored grains.

*F. subglutinans*, which is a member of the *Fusarium fujikuroi* species complex (FFSC), predominantly occurs in temperate climate regions. *F. subglutinans* was elevated to the species level in 1983 by Nelson et al. [11], after separation from *F. verticillioides*. Later on, in 2002, Steenkamp et al. [12] reported two cryptic species within a set of isolates of *F. subglutinans*, obtained from several locations, based on phylogenetic concordance analyses of six nuclear regions, and suggested that both subspecies justify separation into two individual taxa. Phylogenetic separation was further supported by mycotoxin analyses revealing the production of beauvericin (BEA), which was exclusively produced by European isolates belonging to the so-called group 1 of *F. subglutinans* [13]. Furthermore, separation of strains was based on differences in climatic requirements, since isolates of *F. subglutinans* group 1 were frequently collected in cooler regions like Germany, Poland, and Austria while *F. subglutinans* group 2 prevailed in warmer and dryer regions such as Slovakia, Italy, and Serbia [13]. Several other studies also reported the detection of mycotoxins, such as beauvericin [14,15] fusaproliferin (FUSA) [16], moniliformin (MON) [17], and rarely fumonisin B1 (FB1) [18], produced by the subgroup of *F. subglutinans*, presumably *F. subglutinans* group 1. In 2011, this cryptic subgroup was classified as a novel species, establishing *F. temperatum* as *species nova*, corresponding to the formerly known group 1 of *F. subglutinans* [10]. The mycotoxin profile of *F. temperatum* is not yet fully clarified. While the production of beauvericin was consistently found in all strains of *F. temperatum*, moniliformin and fumonisins were produced only by a single isolate of *F.temperatum* as reported by Scauflaire et al. [19]. Similarly, production of beauvericin, moniliformin, fusaproliferin, and fumonisins was reported for *F. temperatum* strains from Argentina [20]. Further studies report on *F. temperatum* infection on maize causing seedling blight and root rot [21] and ear rot, as well as head blight on wheat [22].

The aim of the study was to determine the occurrence of *F. temperatum* and *F. subglutinans* on maize ears and stalks in Germany and to assess their pathogenicity relative to each other and to other common *Fusarium* ear rot and head blight pathogens. In addition, the mycotoxin profiles of *F. temperatum* and *F. subglutinans* were compared.

## 2. Results

### 2.1. Occurrence of Fusarium Species on Cobs and Stalks

In 2017 and 2018, ninety-four isolates of *F. temperatum* and eight isolates of *F. subglutinans* (Table S1) were obtained from diseased cobs collected across eight federal states of Germany (Figure S1). *F. temperatum* was isolated from 17% of all analyzed samples, making it the third most often occurring *Fusarium* species following *F. graminearum* (57%) and *F. verticillioides* (22%) in cobs (n = 293) and the fourth most often isolated species on stalks (n = 190) (Table 1). The frequency of *F. temperatum* isolated from ears ranged from 15% in 2017 to 21% in 2018. *F. subglutinans* was only detected in 2% of all analyzed cobs, at two locations, and in 3% of the stalk samples, at one location.

### 2.2. Pathogenicity on Maize Cobs under Field Conditions

Weather conditions in both years of investigation were conducive for the development of *Fusarium* ear rot infection at the five field sites. Disease severity was strongly affected by pathogen species, year, inoculation method, location, and maize variety as well as the interactions of all factors. According to the mean of squares, pathogen species effects had the highest impact on disease severity (DS), followed by variety and the interaction of both (Table S2). On the average of field experiments, *F. temperatum* was the most aggressive species (31% DS), followed by *F. graminearum* (20% DS), *F. subglutinans* (15% DS), and *F. verticillioides* (11% DS) (Table 2). On the average of the four *Fusarium* species tested, disease severity was favored by silk channel inoculation (26%) compared to needle pin inoculation (13%).

### 2.3. Pathogenicity on Maize Stalks under Greenhouse Conditions

Under greenhouse conditions, *F. graminearum*, *Fusarium crookwellense* (syn. *Fusarium cerealis*), *Fusarium culmorum*, *F. temperatum*, and *F. subglutinans* were able to induce necrotic lesions in the stem tissue and on the surface of the stalks after needle pin inoculation. *F. graminearum* was the most aggressive species on stalks, according to the internal and external lesion length, followed by *F. crookwellense* and *F. culmorum*. Severity of infection, recorded as lesion length, was significantly higher with *F. graminearum* than with *F. temperatum* and *F. subglutinans* (Table 3).

All *F. temperatum* and *F. subglutinans* isolates were able to cause typical symptoms of stalk rot in maize after toothpick inoculation on 49-days old plants (Table 4). Necrotic lesions caused by individual isolates significantly differed from the control treatment. Lesion lengths inside the stalk ranged from 15 mm to 45 mm and outside the stalk from 10 mm to 20 mm. Lesions induced by infection with *F. temperatum* were significantly longer than after inoculation with *F. subglutinans*.

### 2.4. Effect of Temperature on Ear Infection

**Figure 1:**
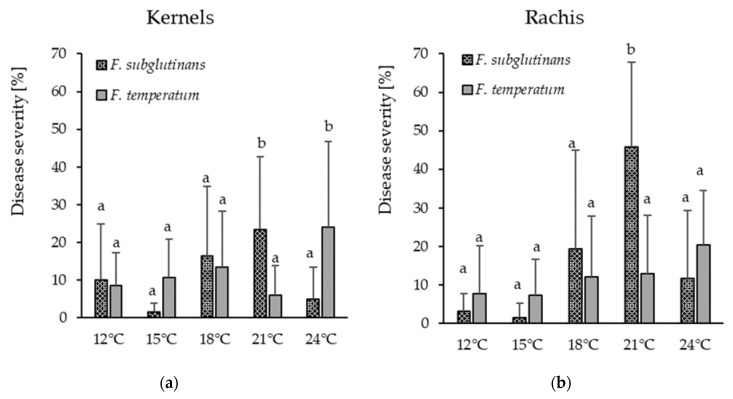
Disease severity induced by *F. subglutinans* and *F. temperatum* at 12 °C, 15 °C, 18 °C, 21 °C, and 24 °C on kernels (**a**) and rachis (**b**) of maize cobs. Vertical bars represent standard deviations. Different letters indicate significant differences (*p* ≤ 0.05).

The temperature had a significant (*p* ≤ 0.05) effect on disease severity of both *F. temperatum* and *F. subglutinans*. The highest contribution to the variance of disease severity originated from the varieties, followed by the interaction of temperature and fungal isolate (Table S3). Inoculation with *F. subglutinans* caused the highest disease severity on kernels (23%) and rachis (46%) at 21 °C. However, the highest disease severity caused by *F. temperatum* on kernels (24%) and rachis (20%) occurred at 24 °C (Figure 1).

### 2.5. Pathogenicity on Wheat under Greenhouse Conditions

All *F. subglutinans* and *F. temperatum* isolates were able to infect winter wheat and to cause symptoms of *Fusarium* head blight (Table 5). The highest disease severity was caused 21 days after infection by *F. graminearum* (52%), followed by *F. temperatum* (44%) and *F. subglutinans* (40%). However, no significant differences were observed between *F. graminearum* and *F. temperatum* regarding disease severity and kernel weight.

### 2.6. Differential Identification of F. temperatum and F. subglutinans

Differential identification of species in the *Fusarium fujikuroi* species complex (FFSC) (*F. temperatum*, *F. subglutinans*, *F. verticillioides*, and *F. proliferatum*,) was performed by species-specific PCR. A total number of 161 single-spore cultures isolated from naturally infected maize cobs and stalk samples in 2018 and grown in PDB were used to extract genomic DNA. The specificity of primers was validated in the studies in which they were designed (references in Table S6). Primers specific for *F. temperatum* allowed amplification of the expected DNA fragments in 44 samples of *F. temperatum*, while samples obtained from pure cultures of *F. subglutinans*, *F. verticillioides*, and *F. proliferatum* did not show amplification. Primers SUB1/SUB2 (Table S7), designed for the amplification of DNA from *F. subglutinans* [23], also generated amplicons in all samples of *F. temperatum* but did not amplify *F. verticillioides* or *F. proliferatum.* Three samples were identified as *F. subglutinans*, as they showed no amplification with primers specific for *F. temperatum* but were positive in the species-specific assay for *F. subglutinans*. Primers for *F. verticillioides* and *F. proliferatum*, the specificity of which has been extensively validated [24], enabled the differential identification of these species in 66 and 48 isolates, respectively.

**Figure 2:**
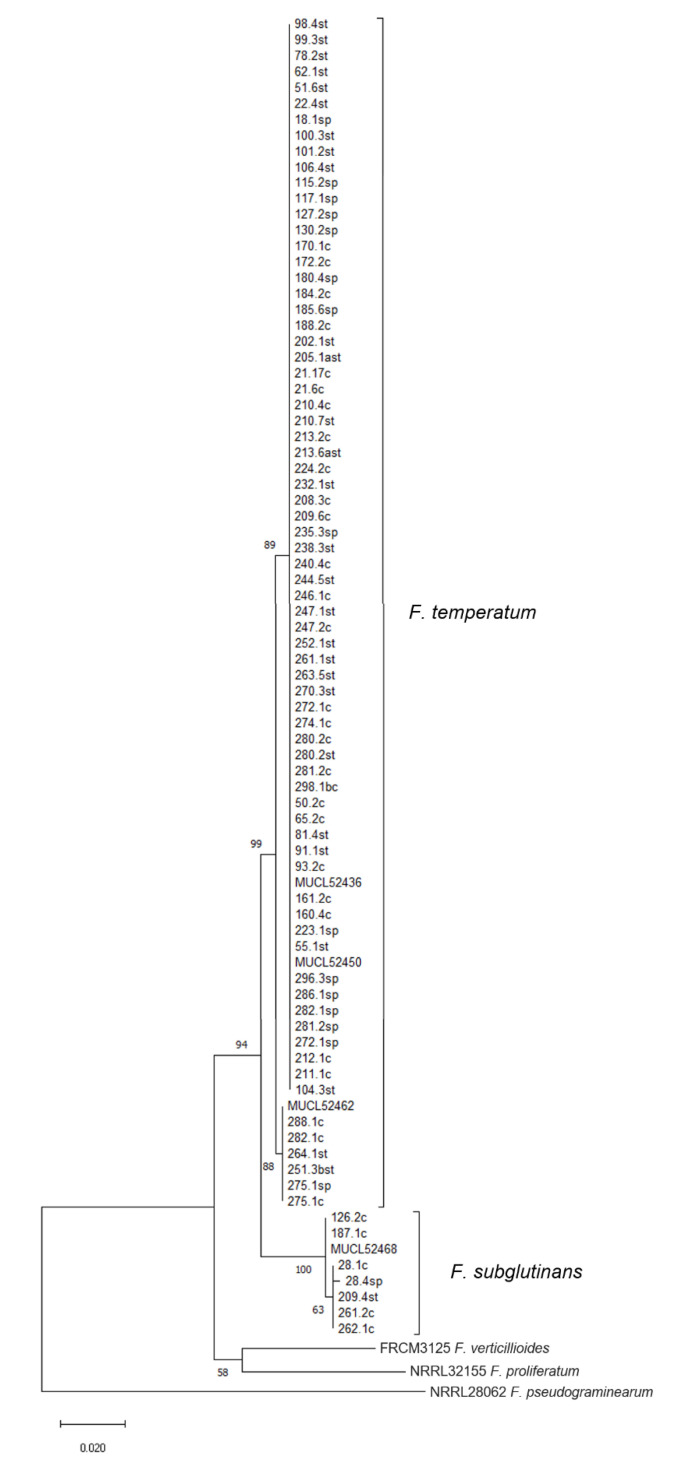
Molecular phylogenetic analysis of translation elongation factor 1 alpha (*TEF-1α*) by the maximum likelihood method (1000 bootstrap replicates) [27]. Analysis was performed with ClustalW [25] in MEGA version 7.0.26 [26] with partial *TEF-1*α sequences of 72 isolates of *F. temperatum* and 7 isolates of *F. subglutinans* (Table S3), and references for *F. temperatum* MUCL52436, MUCL52450, MUCL52462, and *F. subglutinans* MUCL52468 [10]. We added additional references for *F. proliferatum* NRRL32155, *F. verticillioides* FRCM3125, and *F. pseudograminearum* NRRL26062 to scale phylogenetic separation. The tree is drawn to scale, with branch lengths measured in the number of substitutions per site. Bootstrap values are presented next to the nodes. Nucleotide sequences have been subjected to Genbank, the accession numbers are presented in Tables S1 and S5.

Following amplification and sequencing of *TEF-1α*, partial nucleotide sequences (601 bp) from pre-identified isolates of *F. temperatum* and *F. subglutinans* were aligned with additional references of *F. verticillioides*, *F. proliferatum*, and *Fusarium pseudograminearum* using ClustalW [25] in MEGA7 [26] (Figure 2). All sequences of *F. temperatum* (n = 72) and *F. subglutinans* (n = 7) clustered into two groups. The separation between *F. temperatum* and *F. subglutinans* was based on 20 single nucleotide polymorphisms (SNP) within *TEF-1α*. We found 30 isolates of *F. temperatum* with 100% sequence identity with the reference strain MUCL52436, 24 isolates with the same *TEF-1α* sequences as MUCL52450, 5 isolates with *TEF-1α* identical with MUCL52445 and 4 isolates with *TEF-1α* sequence identical with MUCL52454. These sequences formed a separate clade in the phylogenetic tree (Figure 2). Only six samples, identical in their sequence to MUCL52462, were grouped in a further separate clade. The assignments of isolates to individual reference strains are presented in more detail in Table S4. The differentiation among the isolates of *F. temperatum* was mostly based on single SNPs. Thus, despite resampling a relatively uniform population, the phylogenetic analysis showed that isolates of *F. temperatum* can be further divided into two subgroups, which was strongly supported by bootstrap values. Group 1 included the majority of 68 sequences, sharing a high sequence similarity with the reference strains MUCL52436 and MUCL52450. Group 2 is represented by only seven sequences, including MUCL52462. Sequence variation was observed at five positions within the segment of *TEF-1α*, further dividing the species in six genotypes. The grouping within the *F. subglutinans* clade was not supported by bootstrapping.

Analysis of partial *RPB2* was performed for a subset of six isolates, representing the two clades that were observed during investigation of *TEF-1α*. All tested isolates were assigned to a single phylogenetic group, together with reference strain MUCL52463 (Figure S2). Newly obtained sequences were submitted to Genbank; the accession numbers are provided in Tables S1 and S5.

### 2.7. Mycotoxin Analysis

We tested 61 isolates of *F. temperatum* and six isolates of *F. subglutinans*, obtained from 67 naturally infected maize cobs or stalk samples (limited to a single isolate per sample to prevent repeated isolation of the same strains), in cultures of polished rice for the production of the following mycotoxins: fumonisin B1 (FB1), fumonisin B2 (FB2), beauvericin (BEA), fusaric acid (FA), moniliformin (MON), enniatin B (ENNB), enniatin A1 (ENNA1), and fusaproliferin (FUSA). Furthermore, the reference strain MUCL52463 (*F. temperatum*) and isolates of *F. verticillioides* and *F. proliferatum*, identified by sequencing of partial nucleotide sequence of *TEF-1α* (Section 2.6), were included because they have distinct mycotoxin profiles. The results are summarized in Table 6. *F. temperatum* isolates only produced BEA, FA, MON, and FUSA. In *F. subglutinans*, FA, MON, and FUSA but no BEA were detected in all cultures. One isolate of *F. temperatum* and one isolate of *F. subglutinans* produced FB1 and FB2, similarly to *F. verticillioides* and *F. proliferatum*, but the results likely resulted from a contamination because the strains did not possess the fumonisin biosynthetic gene *FUM1* [28] (see below). Rice cultures of *F. verticillioides* and *F. proliferatum* accumulated FB1, FB2, and FUSA, while BEA, FA, and MON were only produced by *F. proliferatum*. No enniatins (ENNB or ENNA1) were detected in any of the analyzed cultures.

### 2.8. Search for FUM1-analogues in F. temperatum and F. subglutinans

The capacity to produce fumonisins was investigated by the amplification and sequencing of a segment of the *FUM1* gene. In a set of isolates, including four isolates of *F. temperatum* and one isolate of *F. subglutinans* that appeared to produce fumonisins *in vitro*, we sequenced the DNA fragment amplified using primers FUM1F1 and FUM1R2, designed by Stepien et al. for the *FUM1* gene [28]. The amplicons were approximately 860 bp long. Amplification of DNA from two randomly chosen isolates of *F. verticillioides* and *F. proliferatum* yielded a single fragment of about 1.1 kb from each isolate. Sequencing and BLAST-analysis revealed the identity of the latter with the gene *FUM1* in *F. verticillioides* (KC188787.1) and *F. proliferatum* (KU180047.1), encoding a polyketide synthase. No similarity at nucleotide or amino acid sequences level could be found between the amplicons generated from *F. temperatum*, *F. subglutinans* DNA with the primers FUM1F1/FUM1R2 [28], and the genomes of *F. verticillioides* or *F. proliferatum*. We also tried out primers Rp32: 5′-ACAAGTGTCCTTGGGGTCCAGG-3′ and Rp33:5′-GATGCTCTTGGAAGTGGCCTACG-3′, specific for the amplification of the *FUM1* gene from *F. verticillioides* [29], but we could not amplify the gene from *F. temperatum* or *F. subglutinans*.

**Figure 3:**
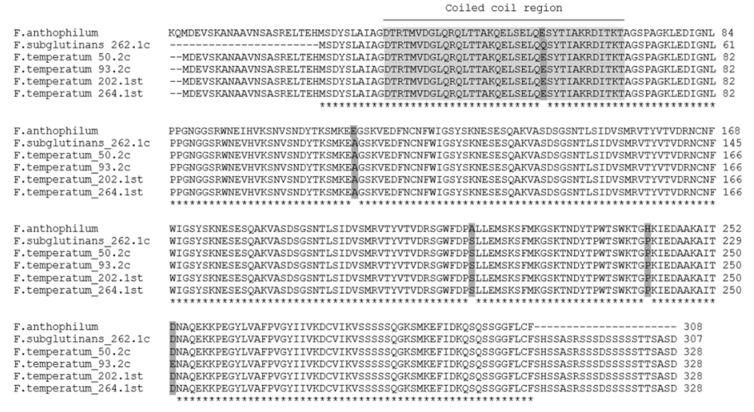
Comparison of the amino acid sequence of a putative *FUM1* analogous gene locus in *F. anthophilum* (FANTH8583), *F. temperatum* (FTEMP8583), and *F. subglutinans* (FSUBG8583). Alignment of amino acid sequences and reference with highest nucleotide sequence similarity after BLASTx search: *F. anthophilum* NRRL25214 (Accession: JABEVY010000206.1). Light grey was used to highlight a coiled coil region, inferred from SMART-analysis [31]. Dark grey highlights amino acid residues differing among the sequences. The alignment was made with ClustalOmega [32]. Symbol * below the alignment indicates identical amino acids residues. Nucleotide sequences were submitted to Genbank; accession numbers are provided in Tables S1 and S5.

Multiple alignment of the sequences revealed high similarity between the genes from *F. subglutinans* and *F. temperatum*, yet no obvious similarity with the *FUM1* gene (Figure 3). Search of NCBI database (BLASTX [30]) with the combined nucleotide data set for four isolates of *F. temperatum* (50.2c, 93.2c, 202.1st, and 264.1st) and one isolate of *F. subglutinans* (262.1c) as a query yielded a single hit in the recently published whole genome shotgun of *Fusarium anthophilum* (strain NRRL 25214). The respective gene of *F. anthophilum* was assigned to a hypothetical protein FANTH_8583. The newly sequenced gene loci in *F. temperatum* and *F. subglutinans*, amplified with primers FUM1F1 and FUM1R2, designed by Stepien et al. [28], were tentatively named FTEMP8583 and FSUBG8583, respectively. Following translation, the amino acid sequences were tested for similarity to known protein domains using the SMART-tool [31]. No similar protein domain was found but a putative coiled coil region was identified. The region started at position 32 and ended at position 68 in the sequences of *F. temperatum*, and started at position 11 and ended at position 47 in the sequences of *F. subglutinans*. The alignment of amino acid sequences is presented in Figure 3. All nucleotide sequences have been subjected to Genbank; the accession numbers are presented in Table S1.

## 3. Discussion

In the monitoring of 2017 and 2018, *F. temperatum* was found to be the third most often occurring *Fusarium* species on maize cobs in Germany. Among all samples, 17% of cob and 17% of stalk samples were infected with *F. temperatum* while only 2% samples were infected with *F. subglutinans*. Similar findings have recently been reported from several European countries, including Poland [33], France [34], and Belgium [10], as well as China [22] and South Korea [35]. 

Environmental factors like precipitation and humidity are known to strongly affect the occurrence and disease severity of several *Fusarium* species [1,11,36,37]. During the two years of monitoring, weather conditions in July differed considerably. In 2017, high precipitation (110.4 mm) occurred and mean temperatures were around 18.8 °C, while in 2018 precipitation was low (40.4 mm) and temperatures were high (20.6 °C). Nonetheless, the frequency of *F. subglutinans* did not change between the years, while the occurrence of *F. temperatum* slightly increased in 2018, suggesting warmer temperatures and low precipitation to be favorable for the latter. Similar tendencies were observed in the inoculation experiments in climate chambers. Disease severity on ears inoculated with *F. temperatum* was highest at 24 °C, while infection with *F. subglutinans* peaked at 21 °C. Even before *F. temperatum* and *F. subglutinans* were defined as individual species, Moretti et al. [13] suggested that a separation into two subgroups may be based on different temperature and humidity requirements, which may have resulted from physiological changes in their sites of origin. Similarly, numerous studies related the occurrence of *F. temperatum* and *F. subglutinans* to temperature and humidity conditions. Moretti et al. [13] observed that *F. subglutinans* occurred more often in warmer and drier regions such as Italy, Slovakia, and Serbia, while *F. temperatum* was reported more often from Germany, Poland, Austria, and Switzerland. These findings correspond to several studies from Belgium [10], China [22] and Argentina [20] indicating higher frequencies of *F. temperatum* in moderate to cool and moist regions with mean temperatures of 18 °C or lower, while other studies reported *F. temperatum* more often in Poland [33] and Germany [38] following dry conditions. Marin et al. [39] demonstrated that the growth rate of most *Fusarium* ssp. increased with increasing water activity (a_w_ value); however, the growth rate of *F. proliferatum* and *F. subglutinans* decreased at 25 °C when the a_w_ value increased from 0.980 to 0.995. Further studies are needed to clarify the effect of temperature and precipitation on the occurrence of and disease incidence caused by *F. temperatum* and *F. subglutinans*.

Field inoculation studies at five locations in Germany in 2017 and 2018 showed *F. temperatum* to be the most aggressive *Fusarium* species in maize, even as compared to *F. graminearum* and *F. verticillioides.* However, no significant differences in pathogenicity were observed between *F. temperatum* and *F. subglutinans* at a field site in 2018. This observation confirms the particular importance of *F. temperatum* as an ear rot pathogen in maize cultivation in Germany and other locations with similar climate. The low visual infection rate of *F. verticillioides* may be explained by symptomless infection and endophytic colonization of maize ears; therefore, disease symptoms may not reflect plant colonization and mycotoxin concentration accurately [40,41,42,43].

Inoculation of maize stalks with toothpicks showed that *F. temperatum* and *F. subglutinans* are pathogenic on the stalks. However, compared to common stalk rot pathogens like *F. graminearum*, *F. culmorum*, and *F. crookwellense*, disease severity was relatively low. This corresponds to the results of Levic et al. [44] and Scauflaire et al. [19], who reported the formation of necrotic lesions and symptoms like wilting, stunting, and rotting on stalks and leaf sheaths by *F. temperatum* and *F. subglutinans* yet lower disease severity as compared to *F. crookwellense*, *F. verticillioides*, *F. culmorum*, and *F. graminearum* [21].

Crop residues of maize infected with *Fusarium* spp. are considered a major inoculum source for *Fusarium* diseases in small grain cereals in Europe, such as seedling and root rot at the seedling stage and *Fusarium* head blight during anthesis [45,46]. *Fusarium* head blight of wheat is mainly caused by *F. graminearum*, *F. culmorum*, *F. poae*, *Fusarium tricinctum*, and *Fusarium avenaceum* [47,48,49]. In addition, *F. subglutinans* was reported to rarely infect wheat, causing contamination with MON in small grain cereals from central to north-east European countries [48,50]. *F. proliferatum* occasionally infects wheat, causing contamination with fumonisins and BEA [51,52]. In our study, all *F. temperatum* and *F. subglutinans* isolates were able to infect winter wheat and cause *Fusarium* head blight at anthesis. The severity of disease caused by *F. graminearum* was highest and the colonization of the plant advanced with the highest rate, while 21 days post infection no significant differences between *F. temperatum* and *F. graminearum* were found with regard to disease severity and thousand-kernel weight. In line with our results, the investigations of Wang et al. [22] demonstrated pathogenicity of *F. temperatum* and *F. subglutinans* on wheat; however, the aggressiveness of tested strains was significantly lower than the aggressiveness of a control strain of *Fusarium asiaticum.*

Identification of *F. temperatum* and *F. subglutinans* was carried out by species-specific PCR assays [23,53] and strengthened by the analysis of the marker gene *TEF-1α*, as previously reported [10]. Phylogenetic analysis enabled the assignment of all obtained isolates to their respective species, as supported by bootstrap values. Separation of isolates and references suggest a rather uniform population of *F. temperatum*, when compared with phylogenetic investigations recently published [9,34,36,54,55,56]. The analysis showed that the isolates of *F. temperatum* are genetically divided into two groups, as supported by a high bootstrap values. This has also been reported by Shin et al. [55] for isolates of *F. temperatum* from Korea, even though the isolates were obtained from a single location. We were unable to link this grouping to morphological characteristics nor to mycotoxin profiles of the respective isolates. The analysis of *TEF-1α* is highly recommended for taxonomical identification of species in the genus *Fusarium* but reliable separation shall be verified by the investigation of additional informative loci [57]. Hence, we selected the gene for the DNA-directed RNA polymerase II subunit (*RPB2*) for verification of distinct groups that we observed in the analysis of *TEF-1α.* The formation of clades could not be reproduced with *RPB2* amplified according to Lofgren et al. [58], using a 763 bp portion of the gene located at the 5′ end. The associated phylogram, based on multiple sequence alignments for a small set of isolates and reference strains, is provided in Figure S2. Therefore, we assume that the grouping of nucleotide sequences of *TEF-1α* does not reflect the genetic relatedness among the strains.

In order to evaluate the risk of mycotoxin contamination upon infection with *F. temperatum* and *F. subglutinans*, in vitro cultures were screened for the presence of eight mycotoxins (BEA, MON, FA, FUSA, ENNB, ENNA1, FB1, and FB2), selected according to Scauflaire et al. [19] and others [14,17,18]. We detected BEA in 58 cultures exclusively of *F. temperatum*, supporting the suitability of BEA production for the separation of *F. temperatum* from *F. subglutinans*, similarly as BEA production separated *F. verticillioides* from *F. proliferatum* [24]. The production of MON, FA, and FUSA was confirmed in almost all tested cultures of *F. temperatum* and *F. subglutinans*. Previous studies suggested that MON may not be produced universally by *F. temperatum* [19]. Based on our results, both *F. temperatum* and *F. subglutinans* produced MON but the amounts varied among isolates. The production of MON and FA by some isolates was so low that it could escape detection. As we found only single isolates showing low production of MON and FA, further studies need to be conducted to clarify this finding. The amounts of FA were low, as reported from other studies [59]. Even though FA exerts low toxicity at levels normally detected in natural infections, synergistic effects have been reported between DON and FA in pigs and FB1 and FA in chicken eggs [60]. Although DON is not produced by the species of the FFSC, both DON and FB1 are common in maize grains and contamination with multiple mycotoxins may occur. FA thus potentially increases the risk of mycotoxin exposure via maize consumption. 

In the present study, all strains of *F. temperatum* and *F. subglutinans* were FUSA-producers. Even though contamination with FUSA and also BEA are rarely reported in literature [61], a significant role of these toxins in the natural toxicity of the producing species, also in association with other toxins, such as MON, was suggested [16]. The biological activity of FUSA remains to be fully elucidated. We were not able to detect ENNB or ENNA1 in any analyzed rice culture; however, low amounts of ENNB were detected in three maize cobs naturally infected with *F. temperatum*, harvested in 2017 (data not shown). These maize cobs were co-infected with *F. temperatum* and *F. avenaceum*, which was likely responsible for the production of ENNB [6]. Production of any enniatins has shown to be a rare event among isolates of *F. temperatum* so it may not be considered a mycotoxin characteristic of *F. temperatum* [19]. Even though the ability to synthesize enniatins is a common feature of some trichothecene producing species of *Fusarium*, such as *F. avenaceum*, [6,62] it has rarely been observed for any species of the GFSC [13]. Enniatins are less toxic than trichothecenes, such as deoxynivalenol. Their function in pathogenesis on maize is still unknown.

The production of fumonisins has been reported in a few cases for both *F. temperatum* and *F. subglutinans*, even though the associated *FUM*-cluster could not be detected in their genomes. The production of these toxins by both species in maize plants [35] can be explained by spontaneous infection of the plants with other species. Wang at al. [22] reported production of fumonisins by *F. temperarum* but because they analyzed grains from the field rather than axenic cultures and have not described the analytical method adequately, their results have not been considered here. We detected fumonisins in only one culture of *F. temperatum*, likely due to contamination, supporting the current view that *F. temperatum* does not produce fumonisins. FB1 and FB2 were also detected in one culture of *F. subglutinans.* Even though *F. subglutinans* was occasionally reported to produce fumonisins [63], which is a common feature among members of the FFSC, both classical [18] as well as modern studies [64,65,66,67] convincingly showed that *F. subglutinans* does not produce fumonisins. Mycotoxin production found in a small set of cultures of *F. verticillioides* and *F. proliferatum* confirmed the established mycotoxin spectra of these species [59,68,69,70].

Gene clusters required for synthesis of sphinganine-analog metabolites, such as fumonisins, in *Fusarium* spp. are conserved [65,66,67]. Sequence analysis of the gene amplified from *F. subglutinans* and *F. temperatum* with primers for the gene *FUM1* revealed that the product was unrelated to *FUM1*. Interestingly, a gene with a high sequence similarity to the product was found in the genome of the closely related species *F. anthophilum*. The amino acid sequence predicted a coiled coil region (Figure 2), possibly indicating involvement of a hypothetical protein in the regulation of gene expression. *F. anthophilum* is a member of the American clade of the FFSC, which includes fumonisin-nonproducing species *F. temperatum* and *F. subglutinans* and fumonisin-producing species *F. anthophilum* and *Fusarium bulbicola* [65]. The authors assume a combination of loss of the respective genes during species divergence and horizontal gene transfer, leading to the loss or retention of fumonisin synthesis.

The results obtained in the present study indicate a high degree of variability in BEA, MON, FA, and FUSA production among isolates of *F. temperatum*. We found isolates with a comparably low toxicity, producing low amounts of FA, BEA, MON, and FUSA, and highly toxic isolates. Our results support the assumption of lower toxigenic risk due to infection of maize with *F. subglutinans* as compared to *F. temperatum*, especially regarding the production of BEA.

In conclusion, the present investigations indicate that *F. temperatum* occurs more frequently on maize cobs and is more aggressive than previously known, and thus represents an elevated threat of food and feed contamination to growers, processing industries, and consumers. In addition, *F. temperatum* may enhance the risk of head blight on wheat if grown in rotation with maize. 

## 4. Materials and Methods

### 4.1. Fungal Isolation and Cultivation

*Fusarium* isolates were obtained from 293 naturally infected maize cobs and 190 stalk samples, which were collected from silage and grain maize at 72 field sites in Germany in 2017 and 2018. Thirty randomly chosen kernels of each cob were surface sterilized for 10 min with 0.1% silver nitrate and incubated on moist sterile filter paper for two days at room temperature. Afterwards, kernels with outgrowing *Fusarium* mycelium were placed on potato dextrose agar (PDA) [71]. The rachis was cut in nine slices, three from the base, three from the middle part, and three from the tip of the cob. The slices were surface sterilized as described above and placed directly on PDA plates. The stalk samples were cut in nine slices, three from the first nodium, three from the internodium, and three from the second nodium. The samples were surface sterilized and placed on PDA plates. After two days, *Fusarium* mycelium outgrown from the sample was transferred to synthetic low nutrition agar (SNA) [71] to produce single spore cultures. The isolates were stored as single spore cultures on synthetic SNA plates at 4 °C. Reference strains of *Fusarium* (Table S7) were grown at 25 °C in the dark.

### 4.2. Inoculum Preparation

Spore suspension was produced according to Reid et al. [72] in liquid media containing 2 g KH_2_PO_4_, 2 g KNO_3_, 1 g MgSO_4_, 1 g KCL, 1 g glucose, 2 mg FeCl_3_, 0.2 mg MnSO_4_, and 0.2 mg ZnSO_4_ in 1 L of water. A plug of agar medium (PDA or SNA) with a diameter of 1 cm overgrown with mycelium was added to 200 mL of the autoclaved medium in a 500 mL Erlenmeyer flask. The medium was placed on a shaker and shaken slowly for 10 days under near-UV-light (λ = 440–400 nm). The spore suspension was filtered through gauze and spore concentration was determined with a Thoma hemocytometer. For *F. graminearum*, spore density was adjusted to 1 × 10^4^ spores per mL. For *F. temperatum*, *F. subglutinans*, *F. crookwellense*, *F. culmorum*, and *F. verticillioides*, the inoculum was adjusted to a density of 1 × 10^6^ spores per mL.

### 4.3. Pathogenicity Test on Maize Cobs under Field Conditions

The field trials in 2018 and 2019 were located at five locations in Germany and France, i.e., Liesborn (North Rhine-Westphalia, Germany), Bernburg (Saxony, Germany), Kuenzing (Bavaria, Germany), and Rustenhart (Gran Est, France). In 2019, an additional field trial was set up in Goettingen. At each location, maize plants of four susceptible varieties were inoculated by silk channel injection and needle pin stabbing with *F. graminearum*, *F. temperatum*, and *F. verticillioides*. Maize plants in Goettingen were inoculated with *F. subglutinans* instead of *F. verticillioides*. Plants were grown in a randomized complete block design, with 75 cm between rows and 13.3 cm between plants (9 plants/m²) in two repetitions. The primary ear of ten plants per row was inoculated with the pathogen, whereas another ten cobs were inoculated with water (control). The time point of inoculation was determined individually based on the time point of flowering. Silk channel inoculation was performed by a self-refilling syringe (Socorex 173, Ecublens, Swiss) seven days after 50% silk emergence in a row. Two mL of spore suspension were injected into silk channels between the cob tip and the point where silks emerge [72]. Needle pin inoculation was conducted 15 days after silk emergence. Prior to wounding, the four stainless steel needles (18 mm long, 10 mm wide) were dipped into the spore suspension and stabbed in the center of the ear through the husk leaves. At physiological maturity, husk leaves of ten *Fusarium*-inoculated and ten control ears were removed, and disease severity was rated. Disease severity on primary ears was assessed visually as percentage (0–100%) of surface covered with mycelium based on the EPPO Guidelines (PP 1/285) [73]. Ten *Fusarium* inoculated and five water inoculated ears per row were harvested, dried, and shelled (Almaco, IA, USA). Temperature and rainfall data were obtained during the whole vegetation period from a weather station close to the field site (<5 km).

### 4.4. Pathogenicity Test on Maize Stalks under Greenhouse Conditions

Pathogenicity on maize stalk was tested at two plant growth stages in two separate experiments, after seven weeks (BBCH 13) by toothpick inoculation, and at flowering (BBCH 65) by needle pin inoculation. Toothpick inoculation was adapted from Scauflaire et al. [19]. Six wooden toothpicks per treatment were autoclaved (three times for 15 min at 121 °C) and preserved in 15 mL tubes with 5 mL of 2% malt extract broth medium (Merck, Darmstadt, Germany). Afterwards, 1 mL of spore suspension of 13 isolates of *F. temperatum* and seven isolates of *F. subglutinans* was added to the preserved toothpicks. Following inoculation, toothpicks were incubated for two weeks at 23 °C in the dark.

Seeds of one maize hybrid were surface sterilized with 0.1% sodium hypochlorite for 10 min and sown in 12 cm diameter pots filled with a mixture of potting soil, compost, and sand (3/1/1). Pots were placed in growth chambers at 22 °C, 50% relative humidity and a day-/night-light cycle of 14/10 h. After seven weeks, stalks were inoculated by piercing with a toothpick overgrown with *Fusarium* 10 cm above the soil surface. The toothpick was cut at both sides of the stalk surface and the inoculation site sealed with Parafilm^®^. Six plants were inoculated per isolate. After 14 days, plants were collected and the length of necrotic lesions around the inoculation point was measured. Lesion length was measured from the stark surface, then the stalk was cut in two halves and necrosis were measured inside of the stalk.

Pathogenicity testing on maize stalks by needle pin inoculation was conducted at the flowering stage. Maize seeds of four susceptible hybrids were seeded in a mixture of potting soil in 20 cm diameter pots. Pots were placed in the greenhouse at 23 °C at a seasonal day-/night-light cycle. Stalks were inoculated with *F. graminearum*, *F. crookwellense*, *F. culmorum*, *F. subglutinans*, and *F. temperatum* by dipping the needle pin into the spore suspension and stabbing in the middle of the first elongated internode of the stalk. The insertion point was sealed with Parafilm M (VWR International, Darmstadt, Germany). Ten plants per treatment were inoculated in two repetitions. Six weeks (42 dpi) after inoculation, disease severity was assessed as mentioned earlier.

### 4.5. Effect of Temperature on Ear Infection

In order to assess and compare the effect of temperature on the aggressiveness of *F. subglutinans* and *F. temperatum* on maize, a climate chamber trial was performed. Plants of a susceptible maize hybrid were sown in 16 cm diameter pots filled with a mixture of potting soil, compost, and sand (3/1/1) and placed in the greenhouse at seasonal temperature and a day-/night-light cycle until flowering. Plants were inoculated by a syringe (Braun, Melsungen, Germany) with two isolates of *F. temperatum* (50.2c and 22.4st, Table S1 and Table 4), differing in the mycotoxin profile, and one isolate of *F. subglutinans* (28.4sp, Table S1 and Table 4), by silk channel inoculation. Inoculation was carried out ten days after silk emergence by injection of 1 mL spore suspension into the silk channel between the cob tip and the point where silks emerge from the husk. Plants were maintained in separate climate chambers at 12 °C, 15 °C, 18 °C, 21 °C, and 24 °C, with a relative humidity of 70% and day-/night-light cycle of 14/10 h. Experiments were carried out in duplicates; five plants were inoculated with sterile water and served as control. Plants were harvested six weeks after inoculation and disease severity was scored visually as mentioned before.

### 4.6. Pathogenicity Test on Wheat under Greenhouse Conditions

The pathogenicity of *F. temperatum* and *F. subglutinans* in comparison to *F. graminearum* was examined on two highly susceptible and one less susceptible winter wheat variety. Seedlings were vernalized for seven weeks at 4 °C and planted in 7 cm diameter pots filled with potting soil and compost (1/1). Pots were placed in the greenhouse at seasonal temperature and day-/night-light cycle. Plants were inoculated with four isolates of *F. temperatum*, three isolates of *F. subglutinans* and one isolate of *F. graminearum* by spray and point inoculation. Ten plants in two repetitions were inoculated with the pathogen and five plants per variety were inoculated with sterile water, which served as control. Point inoculation was conducted with a syringe (Braun, Melsungen, Germany) by injecting 25 µL of spore suspension into the center of two florets at anthesis. Spray inoculation was conducted at the beginning of anthesis by spraying 2 mL spore suspension (same densities as described above) from two sides on cereal heads. Ears were covered with plastic bags for 48 h/days post inoculation. Severity of infection was scored visually as percentage (0–100%).

### 4.7. DNA Extraction, PCR, Sequencing, and Bioinformatic Analysis

Mycelium was carefully scrubbed from the surface of PDA culture plates, inoculated with *Fusarium* spp. obtained from naturally infected maize cobs or reference strains (Table S4), and incubated at 25 °C in the dark for 5–7 days. DNA was extracted from lyophilized mycelium, using a CTAB-based protocol as described by Brandfass and Karlovsky [74]. Quality and quantity of the extracted DNA were assessed on agarose gels (0.8% (*w*/*v*) in 1 × Tris-acetate-EDTA buffer) stained with ethidium bromide. Gel electrophoresis was carried out for 60 min at 4.6 V/cm. 

Species-specific PCR analysis was performed in a CFX384 Thermocycler (Biorad, Ruedigheim, Germany) in 384-well microplates (SARSTEDT AG & Co. KG, Nuembrecht, Germany) using a total reaction volume of 4 μL. Reactions were composed of 1 μL template DNA or ddH_2_O for negative controls and 3 μL of reaction mixture (Table 7 and ddH_2_O; 0.1X SYBR Green I solution (Invitrogen, Karlsruhe, Germany); 1 mg/mL bovine serum albumin (BSA); 0.025 μL of DNA polymerase (Table 7). Individual cycler conditions are summarized in Table 8. All standards as well as the negative control were amplified in duplicates. Following amplification, melting curves were obtained. Samples were heated to 95 °C for 60 s and cooled to 55 °C for 60 s. Afterwards, the temperature was increased from 55 °C to 69 °C by 0.5 °C per cycle with continuous fluorescence measurement. Fluorescent data were obtained during the annealing phase to construct a melting curve at the end of the assay. The PCR was completed by running a melting curve analysis.

Amplification of partial genes *TEF-1α* (694 bp), *RPB2* (ca. 763 bp), and *FUM1* (1118 bp) were performed in a peqSTAR 96 universal gradient thermocycler (PEQLAB, Erlangen, Germany) using 1:100 (*v*/*v*) dilutions of the DNA extract in a total reaction volume of 25 µL. The *TEF-1α* gene was amplified using the primers EF1 and EF2 (Table 9). Partial *RPB2* region was amplified with the primers RPB*2*-5F2 and RPB*2*-7CR, according to Lofgren et al. [58]. For amplification of the *FUM1* gene, we used the primers FUM1F1 and FUM1R2 (Table 1), originally designed for amplification of *FUM1* sequences in *F. proliferatum* [28]. PCR mixtures were composed of Standard *Taq* reaction buffer (10 mM Tris-HCl, 50 mM KCl, 1.5 mM MgCl_2_, pH 8.3 at 25 °C; NEB), 100 µM of each deoxyribonucleoside triphosphate, 0.3 µM of each primer, 0.62 u HotStart-polymerase (NEB) and 1 µL template DNA solution. Final MgCl_2_ concentration was adjusted to 2 mM. PCR conditions for amplification of *TEF-1α* were: initial denaturation for 30 s at 95 °C; 30 cycles consisting of 30 s at 94 °C, 30 s at 58 °C, and 1 min at 68 °C; and final extension for 5 min at 68 °C. PCR conditions for amplification of *FUM1* were: initial denaturation for 30 s at 95 °C; 35 cycles consisting of 30 s at 94 °C, 30 s at 60 °C, and 90 s at 68 °C; and final extension for 5 min at 68 °C. The PCR cycling conditions for amplification of *RPB2* included an initial denaturation for 30 s at 95 °C; 10 cycles consisting of 30 s at 94 °C, a gradual decrease from 62 °C to 53 °C (−1 °C/cycle) for 40 s, and 1 min at 68 °C; 30 cycles of 30 s at 94 °C, 40 s at 56 °C, and 1 min at 68 °C; and final extension for 5 min at 68 °C. All PCR products were purified and sent for Sanger-sequencing to Macrogen Europe (Amsterdam, The Netherlands). Amplicons generated for the *FUM1* gene were purified from an agarose gel by using the FastgeneTM Gel/PCR Extraction kit (Nippon Genetics Europe GmbH, Düren, Germany). Results were evaluated with Chromas version 2.6.6 (South Brisbane, Australia) and used for comparative BLAST analysis. Multiple sequence alignment was then performed by using ClustalW [25] in MEGA version 7.0.26 [26].

### 4.8. Mycotoxin Extraction and HPLC-Analysis

Rice cultures [24] were inoculated with single-spore isolates (SNA, agar plugs of 0.5 cm diameter) of *F. temperatum*, *F. subglutinans*, *F. verticillioides*, and *F. proliferatum* obtained from naturally infected maize cobs, and references strain MUCL52463 (Table S4), kindly provided by Dr. Jonathan Scauflaire (Earth and Life Institute, Louvain-la-Neuve, Belgium). Controls were inoculated with blank culture medium. Tubes were incubated in the dark for 28 days, at 21 °C. Mycotoxins were extracted in 30 mL acetonitrile/water/acetic acid (84/15/1 (*v*/*v*/*v*)), following evaporation and sample preparation in methanol/water (20/80 (*v*/*v*)) for HPLC-MS/MS, as described elsewhere [77]. 

Toxin quantification was performed on an Agilent 1290 Infinity II HPLC system coupled to an Agilent 6460 QQQ (Agilent Technologies, Waldbronn, Germany). Samples were analyzed on a Phenomenex Kinetex C18 column with a particle size of 2.5 µm, 100 Å pore size and 50 × 2.1 mm (Phenomenex Ltd., Aschaffenburg, Germany). A 12-point calibration ranging from 3.9 to 2000 µg/L was used. Final analysis was performed with MassHunter B.0.8.00 (Agilent, Waldbronn, Germany). The MS/MS transitions, limits of detection (LODs) and limits of quantification (LOQs) are listed in Table S4.

### 4.9. Statistical Analysis

Statistical analysis was conducted using STATISTICA version 13 (Statistica GmbH, Germany). Means of lesion length were estimated for inside and outside of the stalk for each *Fusarium* species and isolates using the non-parametric Kruskal–Wallis ANOVA and Mann–Whitney-U-Test by 5% probability. Disease severity of ears and wheat heads were log (x + 1) transformed to normalized data. Analysis of variance (ANOVA) for field and greenhouse experiments were carried out by Tukey-HSD-test at 5% probability. Thousand-kernel-weight (TKW) was analyzed by ANOVA and Tukey-Test at 5% probability.

## Figures and Tables

**Table 1:** Relative occurrence of *F. temperatum*, *F. subglutinans*, *F. graminearum*, and *F. verticillioides* on naturally infected cobs and stalk samples in the field in 2017 and 2018.

	Cobs					Stalks				
*Fusarium* Species	Infected (%)			Sample Sites		Infected (%)		Sample Sites		
	2017	2018	Mean ^1^	2017	2018	2017	2018	Mean ^1^	2017	2018
	n = 180	n = 113		n = 42	n = 18	n = 110	n = 80		n = 21	n = 14
*F. temperatum*	15	21	**17**	21	15	15	20	**17**	7	13
*F. subglutinans*	2	2	**2**	3	3	1	3	**2**	1	2
*F. graminearum*	71	30	**57**	41	15	81	43	**65**	20	11
*F. verticillioides*	13	39	**22**	11	16	22	16	**19**	14	11

^1^ Mean of the percentage of infections in the years 2017 and 2018.

**Table 2:** Disease severity (%) on maize cobs after inoculation with *F. graminearum*, *F. temperatum*, and *F. verticillioides* in 2018 and 2019 at five locations in Germany and France as well as with *F. subglutinans* in 2019 in Goettingen using needle or silk channel inoculation.

	Disease Severity (%)			
	*F. graminearum*	*F. temperatum*	*F. verticillioides*	*F. subglutinans*
2018	28 ± ^1^ 30 a ^2^ B ^3^	40 ± 34 aC	12 ± 15 aA	-
2019	15 ± 22 bC	25 ± 26 bD	11 ± 20 bA	15 ± 19 B
Needle pin	19 ± 26 aB	19 ± 14 aB	5 ± 8 aA	10 ± 7 aA
Silk channel	22 ± 26 aA	44 ± 37 bB	18 ± 22 bA	20 ± 25 bA
Bernburg	25 ± 28 cB	30 ± 31 bC	9 ± 19 aA	-
Kuenzing	21 ± 25 bcB	37 ± 30 bC	13 ± 19 aA	-
Liesborn	18 ± 22 bB	37 ± 35 bC	11 ± 19 aA	-
Rustenhart	27 ± 29 cB	32 ± 27 bC	12 ± 14 aA	-
Goettingen	14 ± 24 aA	21 ± 27 aB	- ^4^	15 ± 19 A
MEAN	20 ± 26 B	31 ± 31 C	11 ± 18 A	15 ± 19 A

^1^ Plus-minus sign (±) represents variation according to standard deviation. ^2^ Small letters indicate significant differences between treatments (*p* ≤ 0.05). ^3^ Capital letters indicate differences (*p* ≤ 0.05) between *Fusarium* species. ^4^ No data has been collected.

**Table 3:** Lesion length inside the stalk and on the stalk surface of maize at BBCH 80, 35 days post inoculation with *F. graminearum*, *F. crookwellense*, *F. culmorum*, *F. temperatum*, *F. subglutinans*, and control. Different letters indicate significant differences between treatments (*p* ≤ 0.05).

Inoculated Species	Lesion Length inside the Stalk (mm)	Lesion Length on the Stalk Surface (mm)
*F. graminearum*	78.3 ± ^1^ 4.1 c	57.7 ± 2.5 c
*F. crookwellense*	75.5 ± 3.7 bc	43.6 ± 1.6 bc
*F. culmorum*	65.7 ± 3.6 bc	40.9 ± 1.7 b
*F. temperatum*	55.5 ± 1.4 b	38.0 ± 1.3 b
*F. subglutinans*	53.0 ± 1.9 b	32.7 ± 0.9 b
Control	17.7 ± 0.6 a	15.0 ± 1.1 a
MEAN	58 ± 3.4 B	37 ± 2.0 A

^1^ Values after plus-minus sign (±) represents standard deviation.

**Table 4:** Lesion length inside the stalk and on the surface of maize stalks 14 days post inoculation in the greenhouse with eight strains of *F. subglutinans* and fourteen strains of *F. temperatum* compared to a water control. Different letters indicate significant differences between treatments (*p* ≤ 0.05).

Isolate	Lesion Length inside the Stalk (mm)	Lesion Length on the Stalk Surface (mm)
Control		
Water	3.4 ± ^1^ 0.4 aA	3.6 ± 0.4 aA
*F. subglutinans*		
Fs 187.1	15.5 ± 1.1 b	10.3 ± 0.5 b
Fs 262.1	16.3 ± 0.5 abc	10.3 ± 0.5 ab
Fs 126.2	22.9 ± 1.1 bc	13.0 ± 0.8 b
Fs 215.6	24.3 ± 0.5 bc	11.2 ± 0.2 b
Fs 209.4st	25.7 ± 0.8 bc	11.5 ± 0.2 b
Fs 261.2	27.8 ± 1.3 c	11.8 ± 0.2 b
Fs 28.4sp	28.0 ± 1.2 c	20.8 ± 0.6 c
MEAN	22.7 ± 1.5 B	13.1 ± 0.6 B
*F. temperatum*		
Ft 18.5	19.6 ± 1.4 b	8.8 ± 0.4 ab
Ft 22.4st	25.5 ± 1.7 bc	8.8 ± 0.3 ab
Ft 184.2	28.2 ± 1.5 bc	20.8 ± 1.3 c
Ft 106.4st	30.2 ± 1.5 bcd	14.2 ± 0.9 bc
Ft 98.4st	30.4 ± 1.4 bcd	16.7 ± 0.8 bc
Ft 65.2	30.5 ± 1.3 bcd	15.8 ± 0.5 bc
Ft 188.2	30.8 ± 1.5 bcd	17.7 ± 1.2 bc
Ft 91.1st	32.3 ± 1.3 bcd	16.2 ± 0.7 bc
Ft 81.4st	33.3 ± 1.7 bcd	16.1 ± 0.3 bc
Ft 127.2sp	33.2 ± 1.2 bcd	18.8 ± 0.9 c
Ft 99.3st	39.0 ± 1.9 cd	19.9 ± 0.9 c
Ft 100.3st	41.1 ± 1.3 cd	12.9 ± 0.7 bc
Ft 50.2	45.4 ± 1.4 d	20.8 ± 1.0 c
MEAN	32.3 ± 1.6 C	15.9 ± 0.9 C

^1^ Plus-minus sign (±) represents variation according to standard deviation.

**Table 5:** Disease severity and thousand-kernel weight (TKW) of winter wheat inoculated in the greenhouse at flowering stage with three different *Fusarium* species. Different letters indicate significant differences within the columns (*p* ≤ 0.05).

Species	Disease Severity (%)			TKW (g)
	7 dpi ^1^	14 dpi	21 dpi	
Control	3 ± ^2^ 6 a	10 ± 11 a	17 ± 15 a	33 ± 10 a
*F. subglutinans*	7 ± 8 ab	19 ± 15 b	40 ± 23 b	34 ± 7 a
*F. temperatum*	7 ± 6 b	18 ± 12 bc	44 ± 24 bc	33 ± 7 ab
*F. graminearum*	12 ± 11 b	28 ± 18 c	52 ± 29 c	27 ± 5 b

^1^ Days post inoculation (dpi). ^2^ Plus-minus sign (±) represents variation according to standard deviation.

**Table 6:** Mycotoxin production in rice cultures of *F. temperatum*, *F. subglutinans*, *F. verticillioides*, and *F. proliferatum*, isolated from naturally infected maize cobs and stalk samples.

Species	No. of Isolates	Toxins						
		FB1	FB2	BEA	FA ^3^	MON	FUSA ^4^	ENNs ^5^
*F. temperatum* ^6^	60	- ^1^	-	++ ^2^	++	++	yes	-
*F. subglutinans* ^6^	5	-	-	-	+ ^2^	+	yes	-
*F. verticillioides*	4	+++	+++	-	-	-	yes	-
*F. proliferatum*	4	+++	+++	+	+	++	yes	-

^1^ Metabolite could not be detected (values were below LOD, Table S6). ^2^ Average concentration of mycotoxins (FB1, FB2, BEA, MON, ENNB, ENNA1): +++ more than 1 g/kg, ++ 0.1 to 1.0 g/kg, + less than 100 mg/kg. ^3^ Average for FA concentration: +++ more than 10 mg/kg, ++ 1 to 10 mg/kg, + less than 1 mg/kg. ^4^ FUSA was qualitatively evaluated; yes, indicates the presence of FUSA in the tested sample. ^5^ ENNB and ENNA1. ^6^ Fumonisins were detected in a single culture of each *F. temperatum* and *F. subglutinans* (see main text).

**Table 7:** Reaction mixtures for species-specific PCR assays.

Target Species	MgCl_2_ (mM)	Primer (μM)	dNTP ^1^ (μM)	DNA-Polymerase ^2^	Reaction Buffer ^1^
*F. temperatum*	2	0.3	150	HotStart *Taq*	Standard *Taq* ^3^
*F. subglutinans*	3.5	0.3	100	*Taq*	ThermoPol^® 4^
*F. verticillioides*	2.5	0.3	100	*Taq*	ThermoPol^®^
*F. proliferatum*	2	0.3	125	*Taq*	ThermoPol^®^

^1^ deoxyribonucleosides (Bioline, Luckenwalde, Germany). ^2^ purchased from New England Biolabs, Beverly, Massachusetts, USA; ^3^ standard *Taq* reaction buffer (10 mM Tris-HCl, 50 mM KCl, 1.5 mM MgCl_2_, pH 8.3 at 25 °C). ^4^ ThermoPol reaction buffer (20 mM Tris-HCl, 10 mM (NH_4_)_2_SO_4_, 10 mM KCl, 2 mM MgSO_4_, 0.1% Triton^®^ X-100, pH 8.8 at 25 °C).

**Table 8:** Cycler conditions for species-specific PCR assays.

Target Species	Initial Denaturation	Denaturation	Annealing	Extension	No. of Cycles
*F. temperatum*	95 °C, 120 s	94 °C, 30 s	63 °C, 30 s	68 °C, 30 s	35
*F. subglutinans*	95 °C, 120 s	94 °C, 30 s	65 °C, 30 s	68 °C, 40 s	35
*F. verticillioides*	95 °C, 120 s	94 °C, 40 s	62.5 °C, 30 s	68 °C, 40 s	35
*F. proliferatum*	95 °C, 120 s	94 °C, 35 s	64 °C, 30 s	68 °C, 35 s	35

**Table 9:** Primers used in this study.

Name	Sequence (5′-3′)	Gene	Amplicon Length (bp)	Reference
SUB 1	CTGTCGCTAACCTCTTTATCCA	*cal* ^1^	631	[23]
SUB 2	CAGTATGGACGTTGGTATTATATCTAA			
FtempF	AAGACCTGGCGGGC	*TEF-1α*	296	[75]
FtempR	TCAGAAGGTTGTGGCAATGG			
VER 1	CTTCCTGCGATGTTTCTCC	*cal*	578	[23]
VER 2 A	ATTGGCCATTGGTATTATATATCTA			
Fp3-F	CGGCCACCAGAGGATGTG	*igs* ^2^	230	[53]
Fp4-R	CAACACGAATCGCTTCCTGAC			
EF1αF	ATGGGTAAGGARGACAAGAC	*TEF-1α*	694	[76]
EF1αR	GGARGTACCAGTRATCATGTT			
RPB*2*-5F2	GGGGWGAYCAG AAGAAGGC	*RPB2*	1200	[58]
RPB*2*-7CR	CCCATRGCTTGYTT RCCCAT			
FUM1F1	CACATCTGTGGGCGATCC	*FUM1*	1118	[28]
FUM1R2	ATATGGCCCCAGCTGCATA			

^1^ calmodulin gene. ^2^ intergenic spacer of rDNA.

## Supplementary Materials

The following are available online at https://www.mdpi.com/2076-0817/9/11/864/s1. Figure S1: Sampling locations. Figure S2: Molecular phylogenetic analysis of DNA-directed RNA polymerase II subunit (*RPB2*). Table S1: List of isolates of *F. temperatum* and *F. subglutinans* and selected isolates of *F. proliferatum* and *F. verticillioides.* Table S2: Analysis of variance from maize cob inoculation under field conditions. Table S3. Analysis of variance from maize cob inoculation at greenhouse conditions at five different temperatures. Table S4: Sequence variations of partial *TEF-1α* gene in isolates of *F. temperatum*. Reference strains of *Fusarium* used in this study. Table S5: Accession numbers of reference sequences used in phylogenetic analysis of *TEF-1α*, *RPB2*, and *FUM1*. HPLC-MS/MS analysis. Table S6: Specification of HPLC-MS/MS analysis. Table S7: Reference strains of *Fusarium*.
