# Supplementary material for: Occurrence, Pathogenicity, and Mycotoxin Production of Fusarium temperatum in Relation to Other Fusarium Species on Maize in Germany
Source: Pathogens. 2020 Oct 22;9(11):864. doi: 10.3390/pathogens9110864 (PMC7690569; doi:10.3390/pathogens9110864)
Supplement: Supplementary file 1 [file pathogens-09-00864-s001.pdf]

Supplementary Materials:

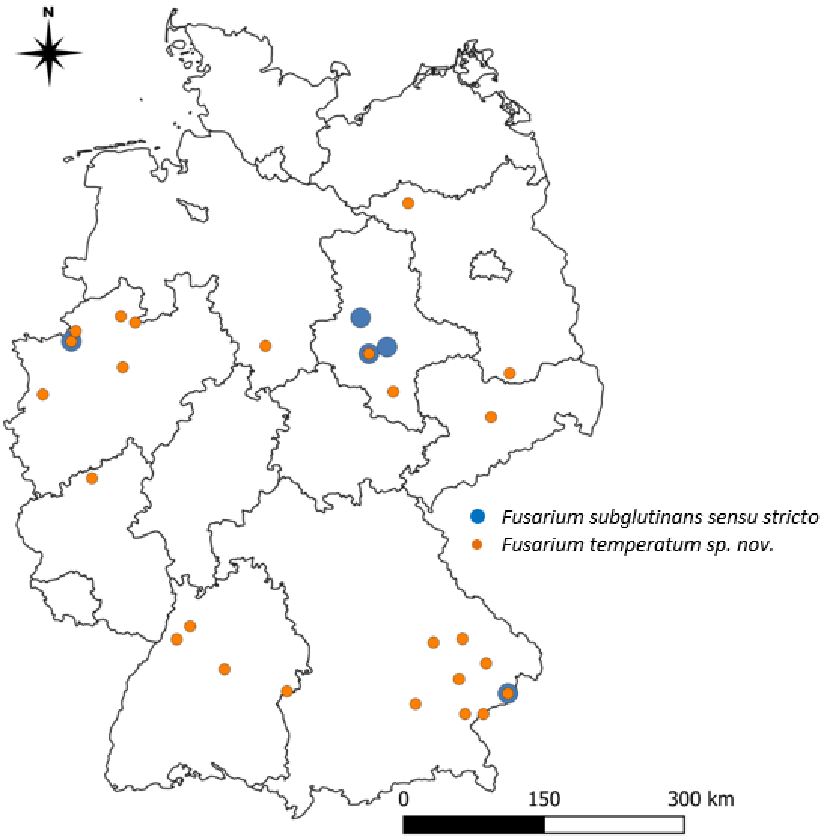

Figure S1. Sampling sites in Germany, where isolates were obtained from maize cobs with infection of *F. subglutinans* and/or *F. temperatum*.

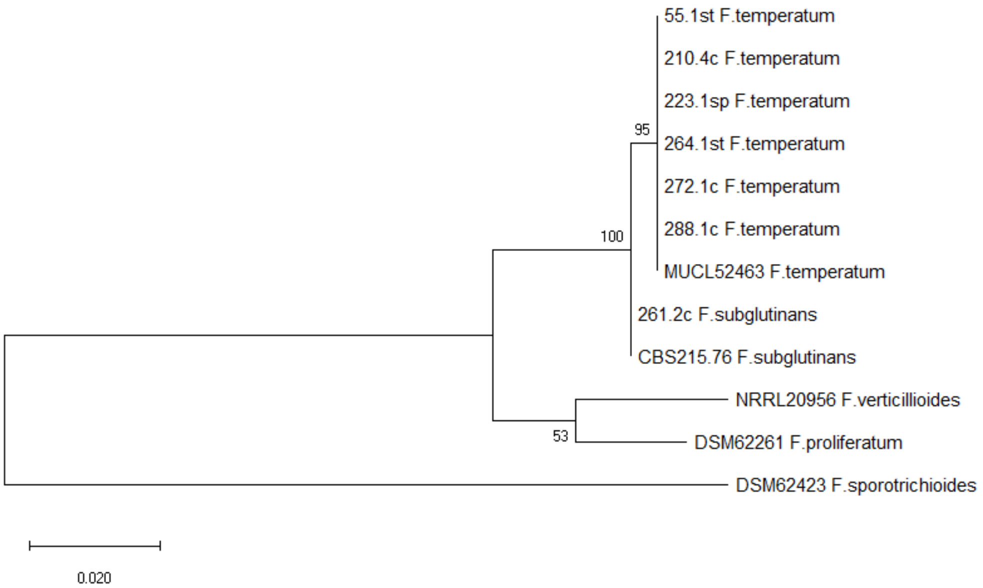

Figure S2. Molecular phylogenetic analysis of DNA-directed RNA polymerase II subunit (*rpb2*) by maximum likelihood method (1000 bootstrap replicates) [25]. Analysis was performed with ClustalW [25] in MEGA version 7.0.26 [26] with partial *rpb2* sequences of 6 isolates of *F. temperatum*, representing phylogenetic group 1 (55.1st, 210.4c, 223.1sp, 272.1c) and group 2 (264.1st, 288.1c) (Figure 1 & Table

S5), 1 isolate of *F. subglutinans,* and references for *F. temperatum* MUCL52463 and *F. subglutinans* CBS215.76 (Table S4). We added additional references DSM62261 *F. proliferatum*, NRRL20956 *F. verticillioides* and DSM62423 *F. sporotrichioides* to scale phylogenetic separation. The tree is drawn to scale, with branch lengths measured in the number of substitutions per site. Bootstrap values are presented next to the nodes. Individual accession numbers are presented in Table S1.

Table S1. List of isolates of *F. temperatum* and *F. subglutinans* and selected isolates of *F. proliferatum*, and *F. verticillioides*, obtained from naturally infected maize cobs, during the present study.

Isolate[1]	Maize organ	Year	Location	Morphological characteristics	Sequencing of *TEF-1α*	Accession numbers		
						TEF-1α	*RPB2*	*FUM1*
Ft 18.1	rachis	2017	Greven	*F. subglutinans* s. lat	*F. temperatum*	MW118553		
Ft 18.5	kernel	2017	Greven	*F. subglutinans* s. lat	*F. temperatum*			
Ft 21.6	kernel	2017	Greven	*F. subglutinans* s. lat	*F. temperatum*	MW118544		
Ft 21.10	kernel	2017	Greven	*F. subglutinans* s. lat	*F. temperatum*			
Ft 22.4	stalk	2017	Gondelsheim	*F. subglutinans* s. lat	*F. temperatum*	MW118536		
Ft 50.2	kernel	2017	Muenzesheim	*F. subglutinans* s. lat	*F. temperatum*	MW118502		MW118590
Ft 51.1	rachis	2017	Muenzesheim	*F. subglutinans* s. lat	*F. temperatum*			
Ft 51.6	stalk	2017	Nossen	*F. subglutinans* s. lat	*F. temperatum*	MW118501		
Ft 55.1	stalk	2017	Nossen	*F. subglutinans* s. lat	*F. temperatum*	MW118500	MW118577	
Ft 61.2	kernel	2017	Pocking	*F. subglutinans* s. lat	*F. temperatum*			
Ft 62.1	stalk	2017	Borken	*F. subglutinans* s. lat	*F. temperatum*	MW118499		
Ft 65.2	kernel	2017	Ostbeven	*F. subglutinans* s. lat	*F. temperatum*	MW118498		
Ft 78.2	stalk	2017	Osterhofen	*F. subglutinans* s. lat	*F. temperatum*	MW118497		
Ft 81.4	stalk	2017	Osterhofen	*F. subglutinans* s. lat	*F. temperatum*	MW118496		
Ft 91.1	stalk	2017	Reith	*F. subglutinans* s. lat	*F. temperatum*	MW118495		
Ft 93.2	kernel	2017	Lauchstaedt	*F. subglutinans* s. lat	*F. temperatum*	MW118494		MW118589
Ft 98.4	stalk	2017	Wesel	*F. subglutinans* s. lat	*F. temperatum*	MW118493		
Ft 99.3	stalk	2017	Wesel	*F. subglutinans* s. lat	*F. temperatum*	MW118492		
Ft 104.3	stalk	2017	Loenningen	*F. subglutinans* s. lat	*F. temperatum*	MW118563		
Ft 100.3	stalk	2017	Wesel	*F. subglutinans* s. lat	*F. temperatum*	MW118565		
Ft 101.2	stalk	2017	Wesel	*F. subglutinans* s. lat	*F. temperatum*	MW118564		
Ft 106.4	stalk	2017	Loenningen	*F. subglutinans* s. lat	*F. temperatum*	MW118562		
Ft 115.2	rachis	2017	Moosham	*F. subglutinans* s. lat	*F. temperatum*	MW118561		
Ft 117.1	rachis	2017	Moosham	*F. subglutinans* s. lat	*F. temperatum*	MW118560		
Ft 127.2	rachis	2017	Borken	*F. subglutinans* s. lat	*F. temperatum*	MW118559		
Ft 130.2	rachis	2017	Westum	*F. subglutinans* s. lat	*F. temperatum*	MW118558		
Ft 160.4	kernel	2017	Osterhoven	*F. subglutinans* s. lat	*F. temperatum*	MW118557		
Ft 161.2	kernel	2017	Osterhoven	*F. subglutinans* s. lat	*F. temperatum*	MW118556		

Ft 170.1	kernel	2017	Wesel	*F. subglutinans* s. lat	*F. temperatum*	MW118555		
Ft 172.2	kernel	2017	Wesel	*F. subglutinans* s. lat	*F. temperatum*	MW118554		
Ft 175.1	kernel	2017	Loenningen	*F. subglutinans* s. lat	*F. temperatum*			
Ft 178.1	kernel	2017	Toenisvorst	*F. subglutinans* s. lat	*F. temperatum*			
Ft 180.4	rachis	2017	Toenisvorst	*F. subglutinans* s. lat	*F. temperatum*	MW118552		
Ft 184.2	kernel	2017	Plessa	*F. subglutinans* s. lat	*F. temperatum*	MW118551		
Ft 185.6	rachis	2017	Plessa	*F. subglutinans* s. lat	*F. temperatum*	MW118550		
Ft 188.2	kernel	2017	Pritzwalk	*F. subglutinans* s. lat	*F. temperatum*	MW118549		
Ft 202.1	stalk	2018	Goettingen	*F. subglutinans* s. lat	*F. temperatum*	MW118548		MW118591
Ft 205.1	stalk	2018	Mintraching	*F. subglutinans* s. lat	*F. temperatum*	MW118547		
Ft 208.2	rachis	2018	Braunau	*F. subglutinans* s. lat	*F. temperatum*			
Ft 208.3	kernel	2018	Braunau	*F. subglutinans* s. lat	*F. temperatum*	MW118546		
Ft 208.5	kernel	2018	Braunau	*F. subglutinans* s. lat	*F. temperatum*			
Ft 208.6	kernel	2018	Braunau	*F. subglutinans* s. lat	*F. temperatum*			
Ft 208.1	rachis	2018	Braunau	*F. subglutinans* s. lat	*F. temperatum*			
Ft 209.6	kernel	2018	Hohenheim	*F. subglutinans* s. lat	*F. temperatum*	MW118545		
Ft 210.4	kernel	2018	Hohenheim	*F. subglutinans* s. lat	*F. temperatum*	MW118543	MW118578	
Ft 210.7	stalk	2018	Pocking	*F. subglutinans* s. lat	*F. temperatum*	MW118542		
Ft 211.1	kernel	2018	Hohenheim	*F. subglutinans* s. lat	*F. temperatum*	MW118541		
Ft 211.1	rachis	2018	Hohenheim	*F. subglutinans* s. lat	*F. temperatum*	MW118540		
Ft 212.1	kernel	2018	Hohenheim	*F. subglutinans* s. lat	*F. temperatum*	MW118539		
Ft 213.2	kernel	2018	Hohenheim	*F. subglutinans* s. lat	*F. temperatum*	MW118538		
Ft 213.6	stalk	2018	Pocking	*F. subglutinans* s. lat	*F. temperatum*	MW118537		
Ft 223.1	rachis	2018	Pocking	*F. subglutinans* s. lat	*F. temperatum*	MW118535	MW118579	
Ft 224.2	kernel	2018	Pocking	*F. subglutinans* s. lat	*F. temperatum*	MW118534		
Ft 224.5	kernel	2018	Pocking	*F. subglutinans* s. lat	*F. temperatum*			
Ft 229.2	rachis	2018	Einbeck	*F. subglutinans* s. lat	*F. temperatum*	MW118533		
Ft 232.1	stalk	2018	Einbeck	*F. subglutinans* s. lat	*F. temperatum*	MW118532		
Ft 235.3	rachis	2018	Frauenberg	*F. subglutinans* s. lat	*F. temperatum*	MW118531		
Ft 238.3	stalk	2018	Frauenberg	*F. subglutinans* s. lat	*F. temperatum*	MW118530		
Ft 240.4	kernel	2018	Ulm Langenau	*F. subglutinans* s. lat	*F. temperatum*	MW118529		
Ft 244.5	stalk	2018	Ulm Langenau	*F. subglutinans* s. lat	*F. temperatum*	MW118528		
Ft 245.1	stalk	2018	Ulm Langenau	*F. subglutinans* s. lat	*F. temperatum*			
Ft 246.1	kernel	2018	Ulm Langenau	*F. subglutinans* s. lat	*F. temperatum*	MW118527		
Ft 247.2	kernel	2018	Ulm Langenau	*F. subglutinans* s. lat	*F. temperatum*	MW118525		

Ft 247.1	rachis	2018	Ulm Langenau	*F. subglutinans* s. lat	*F. temperatum*		
Ft 247.1	stalk	2018	Mintraching	*F. subglutinans* s. lat	*F. temperatum*	MW118526	
Ft 251.3	stalk	2018	Wesel	*F. subglutinans* s. lat	*F. temperatum*	MW118524	
Ft 252.1	stalk	2018	Wesel	*F. subglutinans* s. lat	*F. temperatum*	MW118523	
Ft 261.1	stalk	2018	Toenisvorst	*F. subglutinans* s. lat	*F. temperatum*	MW118522	
Ft 263.5	stalk	2018	Ostbevern	*F. subglutinans* s. lat	*F. temperatum*	MW118521	
Ft 264.1	stalk	2018	Ostbevern	*F. subglutinans* s. lat	*F. temperatum*	MW118520	MW118580 MW118592
Ft 270.3	stalk	2018	Osterhofen	*F. subglutinans* s. lat	*F. temperatum*	MW118519	
Ft 272.1	kernel	2018	Pocking	*F. subglutinans* s. lat	*F. temperatum*	MW118518	MW118581
Ft 272.1	rachis	2018	Pocking	*F. subglutinans* s. lat	*F. temperatum*	MW118517	
Ft 274.1	kernel	2018	Wesel	*F. subglutinans* s. lat	*F. temperatum*	MW118516	
Ft 275.1	kernel	2018	Wesel	*F. subglutinans* s. lat	*F. temperatum*	MW118515	
Ft 275.1	rachis	2018	Wesel	*F. subglutinans* s. lat	*F. temperatum*	MW118514	
Ft 275.3	kernel	2018	Wesel	*F. subglutinans* s. lat	*F. temperatum*		
Ft 280.2	kernel	2018	Toenisvorst	*F. subglutinans* s. lat	*F. temperatum*	MW118513	
Ft 280.4	kernel	2018	Toenisvorst	*F. subglutinans* s. lat	*F. temperatum*		
Ft 280.2	stalk	2018	Altoetting	*F. subglutinans* s. lat	*F. temperatum*	MW118512	
Ft 281.2	kernel	2018	Loeningen	*F. subglutinans* s. lat	*F. temperatum*	MW118511	
Ft 281.2	rachis	2018	Loeningen	*F. subglutinans* s. lat	*F. temperatum*	MW118510	
Ft 282.1	kernel	2018	Loenningen	*F. subglutinans* s. lat	*F. temperatum*	MW118509	
Ft 282.1	rachis	2018	Loeningen	*F. subglutinans* s. lat	*F. temperatum*	MW118508	
Ft 282.2	kernel	2018	Loenningen	*F. subglutinans* s. lat	*F. temperatum*		
Ft 285.1	kernel	2018	Ostbevern	*F. subglutinans* s. lat	*F. temperatum*		
Ft 285.1	rachis	2018	Ostbevern	*F. subglutinans* s. lat	*F. temperatum*		
Ft 286.1	rachis	2018	Ostbevern	*F. subglutinans* s. lat	*F. temperatum*	MW118507	
Ft 287.1	kernel	2018	Ostbevern	*F. subglutinans* s. lat	*F. temperatum*		
Ft 287.1	rachis	2018	Ostbevern	*F. subglutinans* s. lat	*F. temperatum*	MW118506	
Ft 288.1	rachis	2018	Ostbevern	*F. subglutinans* s. lat	*F. temperatum*		
Ft 288.1	kernel	2018	Ostbevern	*F. subglutinans* s. lat	*F. temperatum*	MW118505	MW118582
Ft296.3	rachis	2018	Altoetting	*F. subglutinans* s. lat	*F. temperatum*	MW118504	
Ft 298.1	kernel	2018	Altoetting	*F. subglutinans* s. lat	*F. temperatum*	MW118503	
Fs 28.1	kernel	2017	Bernburg	*F. subglutinans* s. lat	*F. subglutinans* s.str.	MW118567	
Fs 28.4	rachis	2017	Bernburg	*F. subglutinans* s. lat	*F. subglutinans* s.str.	MW118566	
Fs 126.2	kernel	2017	Borken	*F. subglutinans* s. lat	*F. subglutinans* s.str.	MW118572	
Fs 187.1	kernel	2017	Kleinwanzleben	*F. subglutinans* s. lat	*F. subglutinans* s.str.	MW118571	
Fs 209.4	stalk	2018	Pocking	*F. subglutinans* s. lat	*F. subglutinans* s.str.	MW118570	

Fs 215.6	kernel	2018	Kleinwanzleben	*F. subglutinans* s. lat	*F. subglutinans* s.str.			
Fs 261.2	kernel	2018	Mintraching	*F. subglutinans* s. lat	*F. subglutinans* s.str.	MW118569	MW118583	
Fs 262.1	kernel	2018	Pocking	*F. subglutinans* s. lat	*F. subglutinans* s.str.	MW118568		MW118588
Fp 55.9	kernel	2017	Pocking	*F. proliferatum*	*F. proliferatum*	MW118573		
Fp 201.1	kernel	2018	Mintraching	*F. proliferatum*	*F. proliferatum*			MW118594
Fp 209.2	stalk	2018	Pocking	*F. proliferatum*	*F. proliferatum*			
Fp 239.6	kernel	2018	Grucking	*F. proliferatum*	*F. proliferatum*			
Fp 273.1	stalk	2018	Osterhofen	*F. proliferatum*	*F. proliferatum*	MW118574		
Fv 39.4	kernel	2017	Neupotz	*F. verticillioides*	*F. verticillioides*	MW118575		
Fv 205.9	kernel	2018	Mariaposching	*F. verticillioides*	*F. verticillioides*	MW118576		
Fv 208.3	kernel	2018	Braunau	*F. verticillioides*	*F. verticillioides*			MW118593
Fv 236.2	stalk	2018	Grucking	*F. verticillioides*	*F. verticillioides*			
Fv 263.2	kernel	2018	Pocking	*F. verticillioides*	*F. verticillioides*			

[1] Abbreviations of species: Ft = *F. temperatum*, Fs = *F. subglutinans*, Fp = *F. proliferatum*, and Fv = *F. verticillioides*.

Table S2. Multiple variance analyses (year, location, method, variety) and interactions on disease severity of *Fusarium* species on maize cobs under field conditions in 2018 and 2019.

Effect	Degr. of Freedom	F-value	p-value
Year	1	68,751	0,000000
Location	3	32,382	0,000000
Method	1	318,813	0,000000
Variety	19	114,409	0,000000
Year x Location	3	11,160	0,000000
Year x Method	1	15,651	0,000076
Location x Method	3	97,979	0,000000
Year x Variety	19	12,207	0,000000
Location x Variety	57	4,285	0,000000
Method x Variety	19	43,685	0,000000
Year x Location x Method	3	21,903	0,000000
Year x Location x Variety	57	3,876	0,000000
Year x Method x Variety	19	9,093	0,000000
Location x Method x Variety	57	4,942	0,000000
Year x Location x Method x Variety	57	3,048	0,000000
Error	33705		

Table S3. Multiple variance analyses (temperature, isolate, variety) and interactions on disease severity of *F. temperatum* and *F. subglutinans* on maize cobs under greenhouse conditions at five different temperatures.

	Effect on kernels infection			Effect on rachis infection		
**Effect**	**SS**	**FG**	**p-value**	**SS**	**FG**	**p-value**
Temperature	4,3380	4	0,008151	18,4010	4	0,000000
Isolate	4,2537	2	0,000892	2,8193	2	0,010587
Variety	5,3634	1	0,000035	5,9246	1	0,000016
Temperature*Isolate	6,8303	8	0,005027	5,6802	8	0,019797
Temperature*Variety	1,4350	4	0,313567	2,7728	4	0,061330
Isolate*Variety	2,4368	2	0,018560	1,4574	2	0,093004
Temperature*Isolate*Variety	2,0514	8	0,555614	2,8282	8	0,321599

Table S4. Sequence variations of partial *TEF-1α* gene in isolates of *F. temperatum*.

TEF-1α Genotypes	Isolates	SNPs Position[2]	SNPs Nucleotide	Identical reference and accession[3]	Phylogenetic group[4]
1	28[1]	136	G	MUCL52436	1
		142	A	HM067684	
		325	A		
		390	C		
		455	T		
		550	C		
2	3	136	A	MUCL52454	1
		142	G	HM067689	
		325	A		
		390	C		
		455	T		
		550	C		
3	6	136	A	MUCL52462	2
		142	G	HM067690	
		325	T		
		390	C		
		455	A		
		550	T		
4	5	136	A	MUCL52445	1

		142	G	HM067686	
		325	A		
		390	A		
		455	T		
		550	C		
5	24	136	A	MUCL52450	1
		142	A	HM067687	
		325	A		
		390	C		
		455	T		
		550	C		

[1]Three isolates were excluded here, due to low coverage at nucleotide position 136, but matched the first genotype at further SNP positions. [2]Nucleotide Positions in 705 bp PCR product after amplification with EF-1αF and EF-1αR, and sequencing with EF-1αF. [3]Selected reference were described by Scauflaire et al., 2011. [4]Phylogenetic groups 1 and 2 were defined according to the phylogenetic tree presented in Figure 2.

Table S5. Accession numbers for reference strains of *Fusarium* used for phylogenetic analysis of *TEF-1α*, *RPB2* and *FUM1*.

Gene	Species	Isolate/Strain	Accession number[1]
TEF-1α	*F. temperatum*	MUCL52436	HM067684.1
TEF-1α	*F. temperatum*	MUCL52450	HM067687.1
TEF-1α	*F. temperatum*	MUCL52462	HM067690.1
TEF-1α	*F. subglutinans*	MUCL52468	HM067691.1
TEF-1α	*F. proliferatum*	NRRL 32155	FJ538242.1
TEF-1α	*F. verticillioides*	FRC M-3125	KF466424.1
TEF-1α	*F. pseudograminearum*	NRRL28062	AF212468.1
RPB2	*F. verticillioides*	NRRL20956	MN193901.1
RPB2	*F. proliferatum*	DSM62261	MW118586
RPB2	*F. temperatum*	MUCL52463	MW118585
RPB2	*F. subglutinans*	CBS215.76	MW118584
RPB2	*F. sporotrichioides*	DSM62432	MW118587
FUM1	*F. anthophilum*	NRRL25214	JABEVY010000206.1

[1]Nucleotide sequences were obtained from NCBI Genbank.

Table S6. Specification of HPLC-MS/MS analysis.

Toxin	Obtained from	Molecular ion	Parent ion	Product ions	LOD[A] [mg/kg]	LOQ[A] [mg/kg]
BEA	Merck (Darmstadt, Germany)	[M+H]+	784.4	244.0<br>262.2	2.5	20
MON	Enzo Life Sciences (Lörrach, Deutschland)	[M+H]+	97	41.1	1	2
FA	Enzo Life Sciences (Lörrach, Deutschland)	[M+H]+	180.1	162.1<br>134.1	0.02	0.1
FUSA	Dr. Franz Berthiller (BOKU, Vienna, Austria)	[M+H]+	445.3	367.3<br>385.4	-[B]	-
FB1	Merck (Darmstadt, Germany)	[M+H]+	722.4	352.2<br>334.2	3	6
FB2	Enzo Life Sciences (Lörrach, Deutschland)	[M+H]+	706.4	318.3<br>336.3	5	10
ENNA1	Merck (Darmstadt, Germany)	[M+H]+	668.4	228.2<br>210.2	1,5	2
ENNB	Merck (Darmstadt, Germany)	[M+H]+	640.4	527.3<br>196.1	1	2,5

[A]LOD and LOQ were estimated according to blank samples of polished rice, inoculated with pure culture medium. [B]No LOD and LOQ were estimated for fusaproliferin.

Table S7. Reference strains of *Fusarium* used in this study.

Strain ID[1]	Formae specials	Isolated from	Country of origin
NRRL13383	*F. graminearum* Schwabe	*Zea mays*	Iran
DSM62261	*F. proliferatum* (Matsushima) Nirenberg	*Cymbidium* hybrid	Germany
DSM62423	*F. sporotrichioides* Sherbakoff	*Pinus nigra*	Germany
CBS215.76	*F. subglutinans* (Wollenw. & Reinking)	*Zea mays*	Germany
MUCL52463	*F. temperatum* Scauflaire J. & Munaut F.	*Zea mays*	Belgium
NRRL20956	*F. verticillioides* (Saccardo) Nirenberg	*Zea mays*	USA

[1]Fungal strains were obtained from German Collection of Microorganisms and Cell Cultures, Braunschweig, Germany (DSM); Westerdijk Fungal Biodiversity Institute, Baarn, Holland (CBS); ARS culture collection, Peoria, IL, USA (NRRL); and Dr Jonathan Scauflaire, Earth and Life Institute, Louvain-la-Neuve, Belgium (MUCL).
